# The genome sequence of a carabid beetle, *Abax parallelepipedus *(Piller & Mitterpacher, 1783)

**DOI:** 10.12688/wellcomeopenres.23888.1

**Published:** 2025-03-19

**Authors:** Olga Sivell, Duncan Sivell, Ryan Mitchell, Maxwell V.L. Barclay, Natural History Museum Genome Acquisition Lab, Darwin Tree of Life Barcoding collective, Wellcome Sanger Institute Tree of Life Management, Samples and Laboratory team, Wellcome Sanger Institute Scientific Operations: Sequencing Operations, Wellcome Sanger Institute Tree of Life Core Informatics team, Tree of Life Core Informatics collective, Darwin Tree of Life Consortium

**Affiliations:** 1 Natural History Museum, London, England, UK; 2 Independent researcher, Sligo, County Sligo, Ireland

**Keywords:** Abax parallelepipedus, carabid beetle, genome sequence, chromosomal, Coleoptera

## Abstract

We present a genome assembly from a female *Abax parallelepipedus* (carabid beetle; Arthropoda; Insecta; Coleoptera; Carabidae). The genome sequence has a total length of 596.99 megabases. Most of the assembly (97.3%) is scaffolded into 18 chromosomal pseudomolecules, including the X sex chromosome. The mitochondrial genome has also been assembled, with a length of 17.7 kilobases.

## Species taxonomy

Eukaryota; Opisthokonta; Metazoa; Eumetazoa; Bilateria; Protostomia; Ecdysozoa; Panarthropoda; Arthropoda; Mandibulata; Pancrustacea; Hexapoda; Insecta; Dicondylia; Pterygota; Neoptera; Endopterygota; Coleoptera; Adephaga; Caraboidea; Carabidae; Harpalinae; Pterostichini; *Abax*; *Abax parallelepipedus* (Piller & Mitterpacher, 1783) (NCBI:txid102642)

## Background

*Abax parallelepipedus* (Piller & Mitterpacher, 1783) is a species from the family Carabidae (ground beetles) and the only species in the genus *Abax* Bonelli, 1810 resident in Britain. *Abax parallelus* (Duftschmid) is an occasional immigrant encountered on the Isles of Scilly and around 20 species occur in the rest of Europe and Anatolia ( Ball *et al.*, 2000; Müller-Motzfeld, 2004).

*Abax parallelepipedus* is a fairly large species measuring 15.8–20.9 mm in length. It is wide, flat and robust. Males are shiny black; females dull. The elytra have the base completely margined, without dorsal punctures, seventh elytral interval keeled behind the toothed shoulder. Wings are absent. Apical tarsomere has few ventral setae ( Hůrka, 1996; Hůrka, 2005; Lindroth, 1974; Luff, 2007). Several subspecies of *A. parallelepipedus* have been described; the central-western European subspecies were revised by Zanella (2016). The British population belongs to the nominate subspecies *A. parallelepipedus parallelepipedus*, which is also the most widespread subspecies; the others are confined to mountainous regions, particularly in Italy ( Anichtchenko *et al.*, 2025).

*Abax parallelepipedus* is a Palaearctic species distributed in most of Europe, mainly central and northern ( Hůrka, 1996; Hůrka, 2005), but extending eastward through Poland, Hungary and Slovakia into Romania and Ukraine, and westward to the Pyrenees, but it is absent from most of Iberia except the northeast, and absent from North Africa ( Bousquet, 2017). There are unpublished records from European Russia. A record from Iran ( Bousquet, 2017) seems questionable. It has been introduced into Eastern Canada ( Bousquet, 1992). This species is common in forests and in damp, well vegetated moorlands, also in gardens, woodland edges, scrub and cultivated land ( Symondson, 1994). It is widespread in most of Britain, except north-east Scotland ( Luff, 2007; NBN Atlas Partnership, 2025). Adults are active from May to September ( Luff, 2007) but the adult stage can last more than a year, so adults can be found hibernating during the winter.

This species is predacious on slugs and other invertebrates ( Symondson, 1993; Symondson & Liddell, 1993). It has been used to control slugs in polytunnels ( Symondson, 1992; Symondson, 1993). Breeding experiments showed that in favourable conditions the life cycle from egg to adult can take as little as 110 days. The female lays up to 570 eggs in her lifetime. They are laid singly into clay soil capsules moulded by the mother, which is an example of parental care, unusual for Coleoptera ( Brandmayr & Zetto-Brandmayr, 1979; Löser, 1972). The capsules provide camouflage in natural conditions of heavy, clayish soil. The species was shown not to lay eggs when kept on peat. However, when females kept on that substrate were provided with small amounts of clay soil, they would oviposit into clay capsules and deposit them in the peat, where they could be easily found ( Symondson, 1994). The larvae feed on various invertebrates including earthworms. Once fully grown the mature larvae build cells in soil, in which they pupate ( Symondson, 1994). This species has been shown to use unpleasant-smelling secretions that include methacrylic and tiglic acids as a defensive mechanism against predators ( Lečić *et al.*, 2014).

The larva, which is also a free-living predator, was described by Shiodte in 1872 (under the synonym *A. ater*); Luff (1993) provided drawings of larvae as well as identification keys to Carabidae larvae of Fennoscandia and Denmark.

The high-quality genome of *Abax parallelepipedus* presented here was sequenced from a single specimen (NHMUK014036911, SAMEA112221950) from Cothill Fen National Nature Reserve, England, collected on 2021-07-25 by Olga Sivell, Duncan Sivell and Ryan Mitchell. It was identified by Duncan Sivell following Luff (2007) and Hůrka (1996). The high-quality genome was sequenced as part of the Darwin Tree of Life Project, a collaborative effort to sequence all named eukaryotic species in the Atlantic Archipelago of Britain and Ireland. It will aid research on genetics and biology of *A. parallelepipedus,* and phylogeny of the family Carabidae, and may reveal whether the British population of this flightless beetle shows any significant genetic differences, as have been revealed in mountain regions of Southern Europe.

## Genome sequence report

### Sequencing data

**Figure 1.**
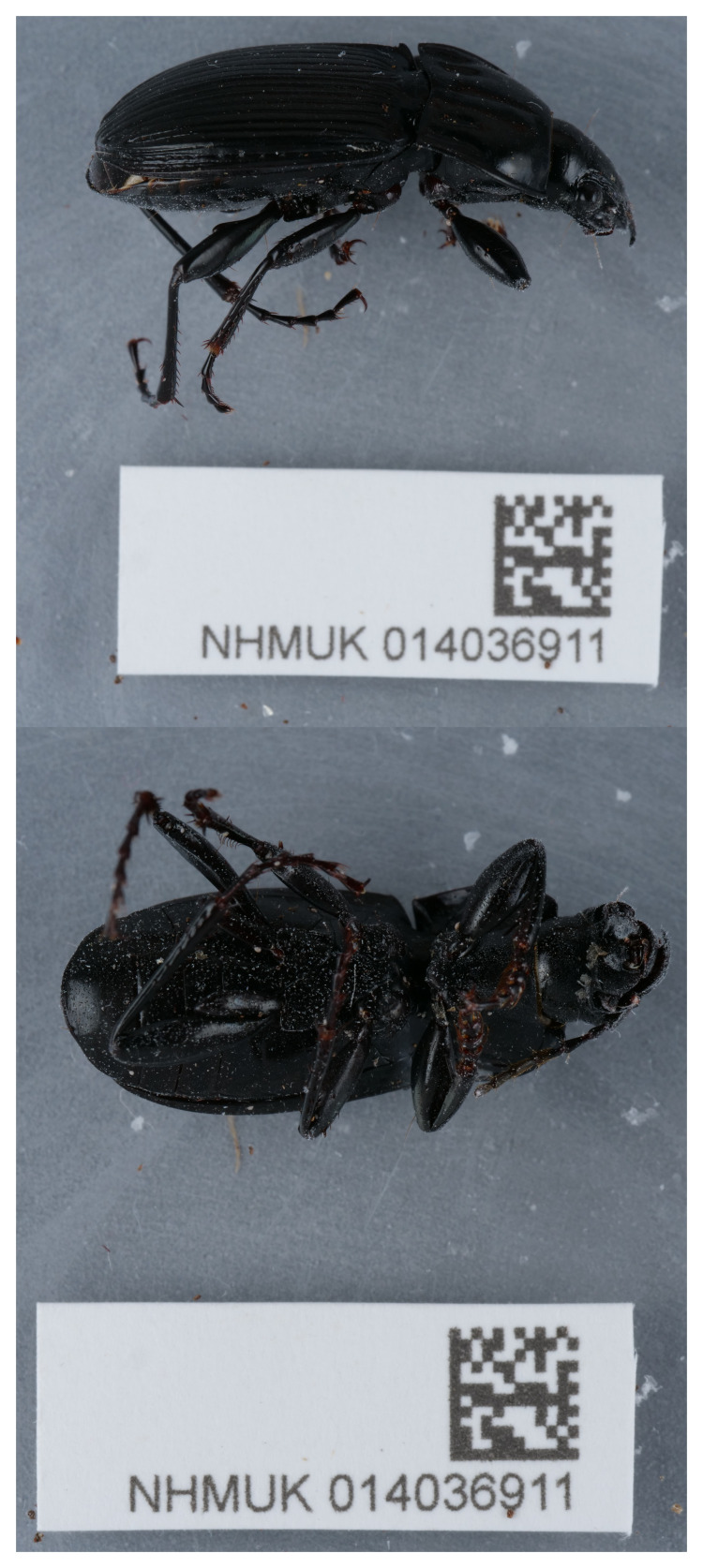
Photograph of the *Abax parallelepipedus* (icAbaPara2) specimen used for genome sequencing.

The genome of a specimen of *Abax parallelepipedus* ( Figure 1) was sequenced using Pacific Biosciences single-molecule HiFi long reads, generating 24.05 Gb (gigabases) from 2.35 million reads. GenomeScope analysis of the PacBio HiFi data estimated the haploid genome size at 567.62 Mb. Based on this estimated genome size, the sequencing data provided approximately 41.0x coverage of the genome. Chromosome conformation Hi-C sequencing produced 199.30 Gb from 1,319.87 million reads. Table 1 summarises the specimen and sequencing information.

**Table 1.** Specimen and sequencing data for *Abax parallelepipedus*.

Project information			
**Study title**	Abax parallelepipedus		
**Umbrella BioProject**	PRJEB64082		
**Species**	*Abax parallelepipedus*		
**BioSpecimen**	SAMEA112221950		
**NCBI taxonomy ID**	102642		
Specimen information			
**Technology**	**ToLID**	**BioSample accession**	**Organism part**
**PacBio long read sequencing**	icAbaPara2	SAMEA112222285	thorax
**Hi-C sequencing**	icAbaPara2	SAMEA112222084	abdomen
**RNA sequencing**	icAbaPara2	SAMEA112222084	abdomen
Sequencing information			
**Platform**	**Run accession**	**Read count**	**Base count (Gb)**
**Hi-C Illumina NovaSeq 6000**	ERR11679403	1.32e+09	199.3
**PacBio Sequel IIe**	ERR11673238	2.35e+06	24.05
**RNA Illumina NovaSeq 6000**	ERR12321228	6.13e+07	9.26

### Assembly statistics

The primary haplotype was assembled, and contigs corresponding to an alternate haplotype were also deposited in INSDC databases. The assembly was improved by manual curation, which corrected 384 misjoins or missing joins and removed 34 haplotypic duplications. These interventions reduced the total assembly length by 0.85%, decreased the scaffold count by 32.33%, and increased the scaffold N50 by 39.8%. The final assembly has a total length of 596.99 Mb in 315 scaffolds, with 553 gaps, and a scaffold N50 of 35.81 Mb ( Table 2).

**Table 2.** Genome assembly data for *Abax parallelepipedus*.

Genome assembly		
Assembly name	icAbaPara2.1	
Assembly accession	GCA_964197645.1	
*Alternate haplotype accession*	*GCA_964197635.1*	
Assembly level for primary assembly	chromosome	
Span (Mb)	596.99	
Number of contigs	868	
Number of scaffolds	315	
Longest scaffold (Mb)	44.93	
Assembly metric	Measure	*Benchmark*
Contig N50 length	3.25 Mb	*≥ 1 Mb*
Scaffold N50 length	35.81 Mb	*= chromosome N50*
Consensus quality (QV)	Primary: 54.6; alternate: 55.0; combined 54.8	*≥ 40*
*k*-mer completeness	Primary: 89.05%; alternate: 73.71%; combined: 98.96%	*≥ 95%*
BUSCO *	C:98.9%[S:98.3%,D:0.6%], F:0.7%,M:0.5%,n:2,124	*S > 90%; D < 5%*
Percentage of assembly mapped to chromosomes	97.08%	*≥ 90%*
Sex chromosomes	X	*localised homologous pairs*
Organelles	Mitochondrial genome: 17.7 kb	*complete single alleles*

* BUSCO scores based on the endopterygota_odb10 BUSCO set using version 5.5.0. C = complete [S = single copy, D = duplicated], F = fragmented, M = missing, n = number of orthologues in comparison.

**Figure 2.**
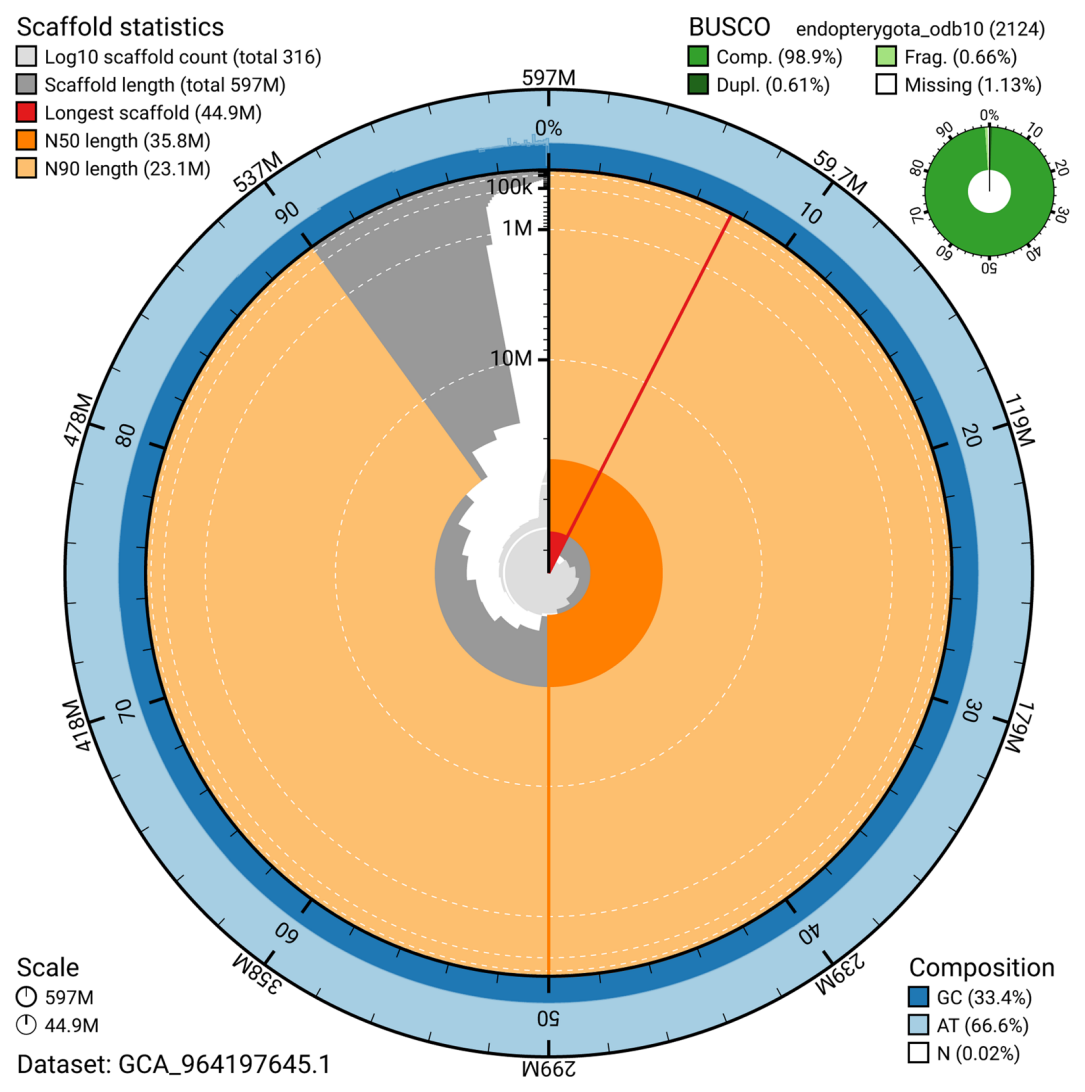
Genome assembly of *Abax parallelepipedus*, icAbaPara2.1: metrics. The BlobToolKit snail plot provides an overview of assembly metrics and BUSCO gene completeness. The circumference represents the length of the whole genome sequence, and the main plot is divided into 1,000 bins around the circumference. The outermost blue tracks display the distribution of GC, AT, and N percentages across the bins. Scaffolds are arranged clockwise from longest to shortest and are depicted in dark grey. The longest scaffold is indicated by the red arc, and the deeper orange and pale orange arcs represent the N50 and N90 lengths. A light grey spiral at the centre shows the cumulative scaffold count on a logarithmic scale. A summary of complete, fragmented, duplicated, and missing BUSCO genes in the endopterygota_odb10 set is presented at the top right. An interactive version of this figure is available at https://blobtoolkit.genomehubs.org/view/GCA_964197645.1/datasets/GCA_964197645.1/snail.

**Figure 3.**
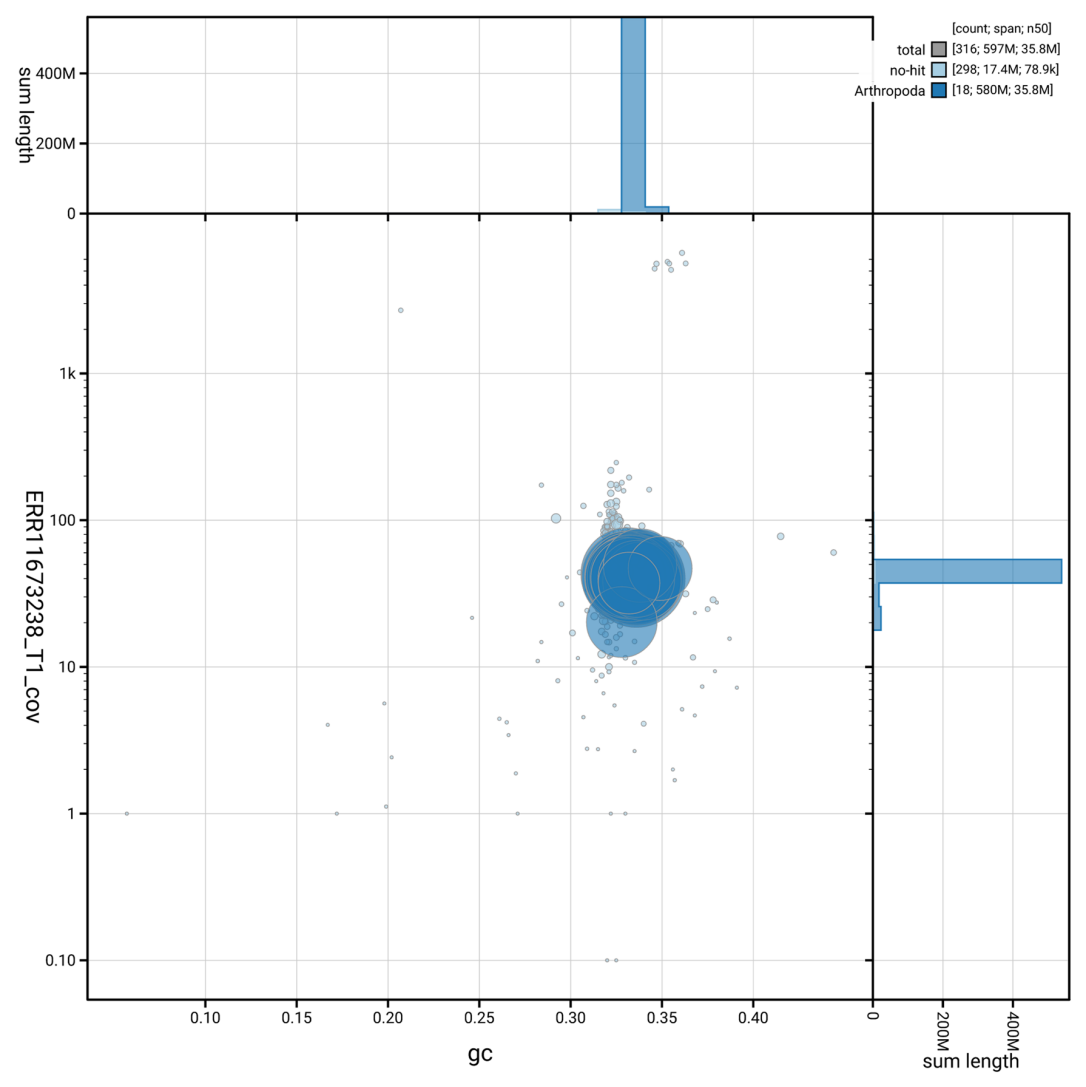
Genome assembly of *Abax parallelepipedus*, icAbaPara2.1: BlobToolKit GC-coverage plot. Blob plot showing sequence coverage (vertical axis) and GC content (horizontal axis). The circles represent scaffolds, with the size proportional to scaffold length and the colour representing phylum membership. The histograms along the axes display the total length of sequences distributed across different levels of coverage and GC content. An interactive version of this figure is available at https://blobtoolkit.genomehubs.org/view/GCA_964197645.1/blob.

**Figure 4.**
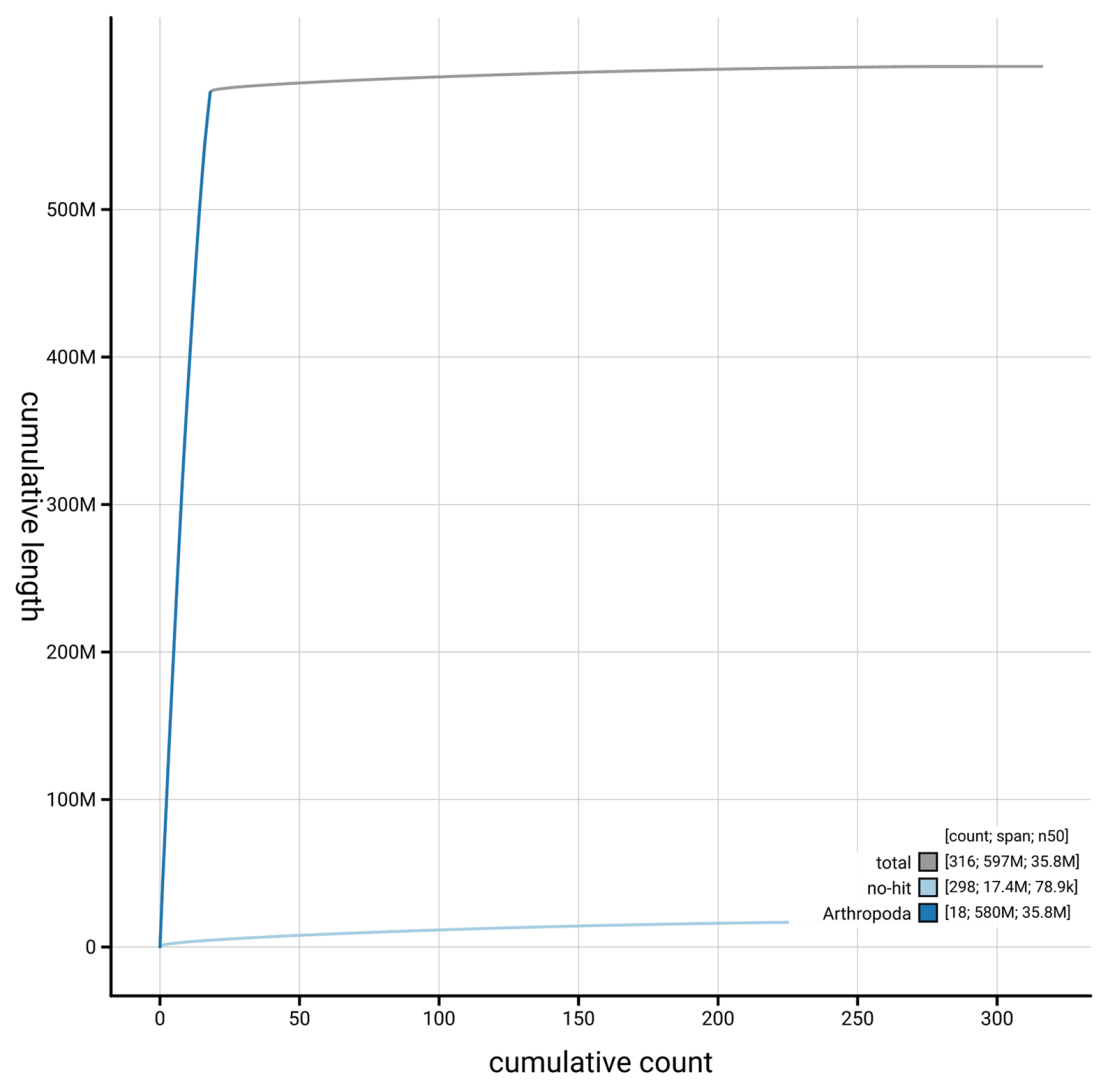
Genome assembly of *Abax parallelepipedus,* icAbaPara2.1: BlobToolKit cumulative sequence plot. The grey line shows cumulative length for all scaffolds. Coloured lines show cumulative lengths of scaffolds assigned to each phylum using the buscogenes taxrule. An interactive version of this figure is available at https://blobtoolkit.genomehubs.org/view/GCA_964197645.1/datasets/GCA_964197645.1/cumulative.

The snail plot in Figure 2 provides a summary of the assembly statistics, indicating the distribution of scaffold lengths and other assembly metrics. Figure 3 shows the distribution of scaffolds by GC proportion and coverage. Figure 4 presents a cumulative assembly plot, with separate curves representing different scaffold subsets assigned to various phyla, illustrating the completeness of the assembly.

**Figure 5.**
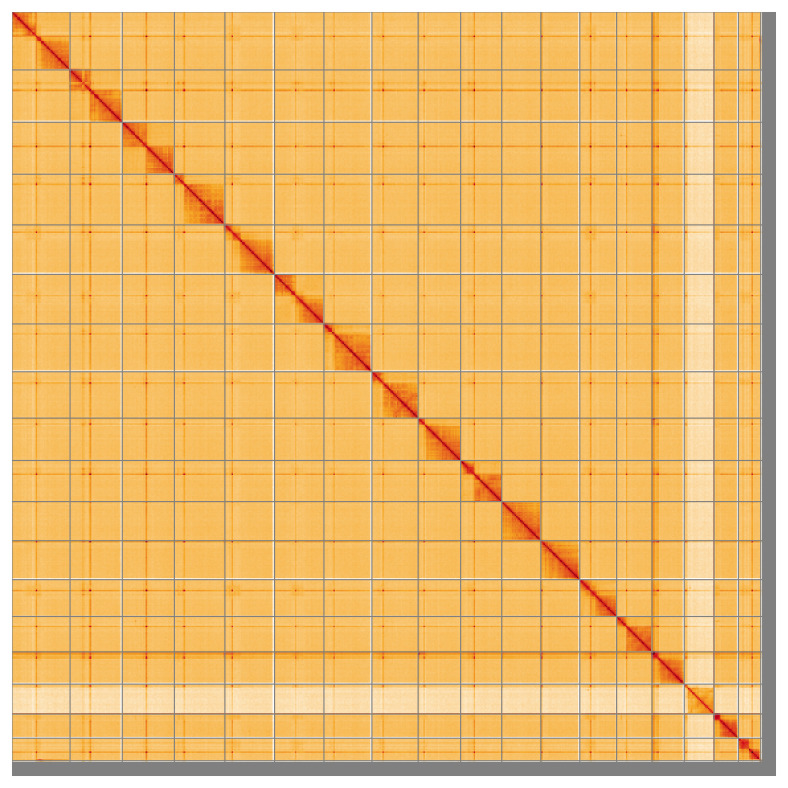
Genome assembly of *Abax parallelepipedus*: Hi-C contact map of the icAbaPara2.1 assembly, visualised using HiGlass. Chromosomes are shown in order of size from left to right and top to bottom. An interactive version of this figure may be viewed at https://genome-note-higlass.tol.sanger.ac.uk/l/?d=J6_XSBc9RI2RSsRG9MIGxg.

Most of the assembly sequence (97.08%) was assigned to 18 chromosomal-level scaffolds, representing 17 autosomes and the X sex chromosome. These chromosome-level scaffolds, confirmed by Hi-C data, are named according to size ( Figure 5; Table 3). During curation, the X chromosome X was assigned by alignment to the genomes of *Pterostichus madidus* (GCA_911728475.2) ( Crowley *et al.*, 2021) and *Pterostichus niger* (GCA_947425015.1) ( Barclay *et al.*, 2023).

**Table 3.** Chromosomal pseudomolecules in the genome assembly of *Abax parallelepipedus*, icAbaPara2.

INSDC accession	Name	Length (Mb)	GC%
OZ078025.1	1	44.93	33.5
OZ078026.1	2	40.49	33
OZ078027.1	3	40.28	33.5
OZ078028.1	4	39.15	33
OZ078029.1	5	38.34	33.5
OZ078030.1	6	38.32	33.5
OZ078031.1	7	37.07	33
OZ078032.1	8	35.81	33.5
OZ078033.1	9	32.87	33
OZ078034.1	10	31.8	33.5
OZ078035.1	11	30.26	33
OZ078036.1	12	30.12	33.5
OZ078037.1	13	28.53	34
OZ078038.1	14	27.39	33.5
OZ078039.1	15	24.97	34
OZ078041.1	16	18.72	35
OZ078042.1	17	17.39	33
OZ078040.1	X	23.14	33
OZ078043.1	MT	0.02	21

The mitochondrial genome was also assembled. This sequence is included as a contig in the multifasta file of the genome submission and as a standalone record.

### Assembly quality metrics

The estimated Quality Value (QV) and *k*-mer completeness metrics, along with BUSCO completeness scores, were calculated for each haplotype and the combined assembly. The QV reflects the base-level accuracy of the assembly, while *k*-mer completeness indicates the proportion of expected *k*-mers identified in the assembly. BUSCO scores provide a measure of completeness based on benchmarking universal single-copy orthologues.

The combined primary and alternate assemblies achieve an estimated QV of 54.8. The *k*-mer recovery for the primary haplotype is 89.05%, and for the alternate haplotype 73.71%; the combined primary and alternate assemblies have a *k*-mer recovery of 98.96%. BUSCO analysis using the endopterygota_odb10 reference set ( *n* = 2,124) identified 98.9% of the expected gene set (single = 98.3%, duplicated = 0.6%).

Table 2 provides assembly metric benchmarks adapted from Rhie *et al.* (2021) and the Earth BioGenome Project Report on Assembly Standards September 2024. The assembly achieves the EBP reference standard of **6.C.Q54.**

## Methods

### Sample acquisition and DNA barcoding

An adult female *Abax parallelepipedus* (specimen ID NHMUK014036911, ToLID icAbaPara2) was collected from Cothill Fen National Nature Reserve, England, United Kingdom (latitude 51.69, longitude –1.32) on 2021-07-25 by handpicking. The specimen was collected by Duncan Sivell, Ryan Mitchell and Olga Sivell (Natural History Museum) and identified by Duncan Sivell (Natural History Museum) and preserved by dry freezing (–80 °C).

The initial identification was verified by an additional DNA barcoding process according to the framework developed by Twyford *et al*. (2024). A small sample was dissected from the specimen and stored in ethanol, while the remaining parts were shipped on dry ice to the Wellcome Sanger Institute (WSI) ( Pereira *et al.*, 2022). The tissue was lysed, the COI marker region was amplified by PCR, and amplicons were sequenced and compared to the BOLD database, confirming the species identification ( Crowley *et al.*, 2023). Following whole genome sequence generation, the relevant DNA barcode region was also used alongside the initial barcoding data for sample tracking at the WSI ( Twyford *et al.*, 2024). The standard operating procedures for Darwin Tree of Life barcoding have been deposited on protocols.io ( Beasley *et al.*, 2023).

Metadata collection for samples adhered to the Darwin Tree of Life project standards described by Lawniczak *et al*. (2022).

### Nucleic acid extraction

The workflow for high molecular weight (HMW) DNA extraction at the Wellcome Sanger Institute (WSI) Tree of Life Core Laboratory includes a sequence of procedures: sample preparation and homogenisation, DNA extraction, fragmentation and purification. Detailed protocols are available on protocols.io ( Denton *et al.*, 2023b). The icAbaPara2 sample was prepared for DNA extraction by weighing and dissecting it on dry ice ( Jay *et al.*, 2023). Tissue from the thorax was homogenised using a PowerMasher II tissue disruptor ( Denton *et al.*, 2023a). HMW DNA was extracted in the WSI Scientific Operations core using the Automated MagAttract v2 protocol ( Oatley *et al.*, 2023). The DNA was sheared into an average fragment size of 12–20 kb in a Megaruptor 3 system ( Bates *et al.*, 2023). Sheared DNA was purified by solid-phase reversible immobilisation, using AMPure PB beads to eliminate shorter fragments and concentrate the DNA ( Strickland *et al.*, 2023). The concentration of the sheared and purified DNA was assessed using a Nanodrop spectrophotometer and Qubit Fluorometer using the Qubit dsDNA High Sensitivity Assay kit. Fragment size distribution was evaluated by running the sample on the FemtoPulse system.

RNA was extracted from abdomen tissue of icAbaPara2 in the Tree of Life Laboratory at the WSI using the RNA Extraction: Automated MagMax™ *mir*Vana protocol ( do Amaral *et al.*, 2023). The RNA concentration was assessed using a Nanodrop spectrophotometer and a Qubit Fluorometer using the Qubit RNA Broad-Range Assay kit. Analysis of the integrity of the RNA was done using the Agilent RNA 6000 Pico Kit and Eukaryotic Total RNA assay.

### Hi-C sample preparation and cross-linking

Tissue from the abdomen of the icAbaPara2 sample was processed for Hi-C sequencing at the WSI Scientific Operations core, using the Arima-HiC v2 kit. In brief, 20–50 mg of frozen tissue (stored at –80 °C) was fixed, and the DNA crosslinked using a TC buffer with 22% formaldehyde concentration. After crosslinking, the tissue was homogenised using the Diagnocine Power Masher-II and BioMasher-II tubes and pestles. Following the Arima-HiC v2 kit manufacturer's instructions, crosslinked DNA was digested using a restriction enzyme master mix. The 5’-overhangs were filled in and labelled with biotinylated nucleotides and proximally ligated. An overnight incubation was carried out for enzymes to digest remaining proteins and for crosslinks to reverse. A clean up was performed with SPRIselect beads prior to library preparation. Additionally, the biotinylation percentage was estimated using the Qubit Fluorometer v4.0 (Thermo Fisher Scientific) and Qubit HS Assay Kit and Arima-HiC v2 QC beads.

### Library preparation and sequencing

Library preparation and sequencing were performed at the WSI Scientific Operations core.

***PacBio HiFi***

At a minimum, samples were required to have an average fragment size exceeding 8 kb and a total mass over 400 ng to proceed to the low input SMRTbell Prep Kit 3.0 protocol (Pacific Biosciences, California, USA), depending on genome size and sequencing depth required. Libraries were prepared using the SMRTbell Prep Kit 3.0 (Pacific Biosciences, California, USA) as per the manufacturer's instructions. The kit includes the reagents required for end repair/A-tailing, adapter ligation, post-ligation SMRTbell bead cleanup, and nuclease treatment. Following the manufacturer’s instructions, size selection and clean up was carried out using diluted AMPure PB beads (Pacific Biosciences, California, USA). DNA concentration was quantified using the Qubit Fluorometer v4.0 (Thermo Fisher Scientific) with Qubit 1X dsDNA HS assay kit and the final library fragment size analysis was carried out using the Agilent Femto Pulse Automated Pulsed Field CE Instrument (Agilent Technologies) and gDNA 55kb BAC analysis kit.

Samples were sequenced using the Sequel IIe system (Pacific Biosciences, California, USA). The concentration of the library loaded onto the Sequel IIe was in the range 40–135 pM. The SMRT link software, a PacBio web-based end-to-end workflow manager, was used to set-up and monitor the run, as well as perform primary and secondary analysis of the data upon completion.

***Hi-C***

For Hi-C library preparation, DNA was fragmented using the Covaris E220 sonicator (Covaris) and size selected using SPRISelect beads to 400 to 600 bp. The DNA was then enriched using the Arima-HiC v2 kit Enrichment beads. Using the NEBNext Ultra II DNA Library Prep Kit (New England Biolabs) for end repair, A-tailing, and adapter ligation. This uses a custom protocol which resembles the standard NEBNext Ultra II DNA Library Prep protocol but where library preparation occurs while DNA is bound to the Enrichment beads. For library amplification, 10 to 16 PCR cycles were required, determined by the sample biotinylation percentage. The Hi-C sequencing was performed using paired-end sequencing with a read length of 150 bp on an Illumina NovaSeq 6000 instrument.

***RNA***

Poly(A) RNA-Seq libraries were constructed using the NEB Ultra II RNA Library Prep kit, following the manufacturer’s instructions. RNA sequencing was performed on the Illumina NovaSeq 6000 instrument.

### Genome assembly, curation and evaluation

***Assembly***

Prior to assembly of the PacBio HiFi reads, a database of *k*-mer counts ( *k* = 31) was generated from the filtered reads using FastK. GenomeScope2 ( Ranallo-Benavidez *et al.*, 2020) was used to analyse the *k*-mer frequency distributions, providing estimates of genome size, heterozygosity, and repeat content.

[primary mode] The HiFi reads were first assembled using Hifiasm ( Cheng *et al.*, 2021) with the --primary option. Haplotypic duplications were identified and removed using purge_dups ( Guan *et al.*, 2020). The Hi-C reads were mapped to the primary contigs using bwa-mem2 ( Vasimuddin *et al.*, 2019). The contigs were further scaffolded using the provided Hi-C data ( Rao *et al.*, 2014) in YaHS ( Zhou *et al.*, 2023) using the --break option for handling potential misassemblies. The scaffolded assemblies were evaluated using Gfastats ( Formenti *et al.*, 2022), BUSCO ( Manni *et al.*, 2021) and MERQURY.FK ( Rhie *et al.*, 2020).

[Hi-C phasing mode] The HiFi reads were assembled using Hifiasm in Hi-C phasing mode ( Cheng *et al.*, 2021; Cheng *et al.*, 2022), resulting in a pair of haplotype-resolved assemblies. Haplotypic duplications were identified and removed using purge_dups ( Guan *et al.*, 2020). The Hi-C reads were mapped to the primary contigs using bwa-mem2 ( Vasimuddin *et al.*, 2019). The contigs were further scaffolded using the provided Hi-C data ( Rao *et al.*, 2014) in YaHS ( Zhou *et al.*, 2023) using the --break option for handling potential misassemblies. The scaffolded assemblies were evaluated using Gfastats ( Formenti *et al.*, 2022), BUSCO ( Manni *et al.*, 2021) and MERQURY.FK ( Rhie *et al.*, 2020).

The mitochondrial genome was assembled using MitoHiFi ( Uliano-Silva *et al.*, 2023), which runs MitoFinder ( Allio *et al.*, 2020) and uses these annotations to select the final mitochondrial contig and to ensure the general quality of the sequence.

***Assembly curation***

The assembly was decontaminated using the Assembly Screen for Cobionts and Contaminants (ASCC) pipeline. Flat files and maps used in curation were generated via the TreeVal pipeline ( Pointon *et al.*, 2023). Manual curation was conducted primarily in PretextView ( Harry, 2022) and HiGlass ( Kerpedjiev *et al.*, 2018), with additional insights provided by JBrowse2 ( Diesh *et al.*, 2023). Scaffolds were visually inspected and corrected as described by Howe *et al*. (2021). Any identified contamination, missed joins, and mis-joins were amended, and duplicate sequences were tagged and removed. The curation process is documented at https://gitlab.com/wtsi-grit/rapid-curation.

***Assembly quality assessment***

The Merqury.FK tool ( Rhie *et al.*, 2020), run in a Singularity container ( Kurtzer *et al.*, 2017), was used to evaluate *k*-mer completeness and assembly quality for the primary and alternate haplotypes using the *k*-mer databases ( *k* = 31) that were computed prior to genome assembly. The analysis outputs included assembly QV scores and completeness statistics.

A Hi-C contact map was produced for the final version of the assembly. The Hi-C reads were aligned using bwa-mem2 ( Vasimuddin *et al.*, 2019) and the alignment files were combined using SAMtools ( Danecek *et al.*, 2021). The Hi-C alignments were converted into a contact map using BEDTools ( Quinlan & Hall, 2010) and the Cooler tool suite ( Abdennur & Mirny, 2020). The contact map was visualised in HiGlass ( Kerpedjiev *et al.*, 2018).

The blobtoolkit pipeline is a Nextflow port of the previous Snakemake Blobtoolkit pipeline ( Challis *et al.*, 2020). It aligns the PacBio reads in SAMtools and minimap2 ( Li, 2018) and generates coverage tracks for regions of fixed size. In parallel, it queries the GoaT database ( Challis *et al.*, 2023) to identify all matching BUSCO lineages to run BUSCO ( Manni *et al.*, 2021). For the three domain-level BUSCO lineages, the pipeline aligns the BUSCO genes to the UniProt Reference Proteomes database ( Bateman *et al.*, 2023) with DIAMOND blastp ( Buchfink *et al.*, 2021). The genome is also divided into chunks according to the density of the BUSCO genes from the closest taxonomic lineage, and each chunk is aligned to the UniProt Reference Proteomes database using DIAMOND blastx. Genome sequences without a hit are chunked using seqtk and aligned to the NT database with blastn ( Altschul *et al.*, 1990). The blobtools suite combines all these outputs into a blobdir for visualisation.

The blobtoolkit pipeline was developed using nf-core tooling ( Ewels *et al.*, 2020) and MultiQC ( Ewels *et al.*, 2016), relying on the Conda package manager, the Bioconda initiative ( Grüning *et al.*, 2018), the Biocontainers infrastructure ( da Veiga Leprevost *et al.*, 2017), as well as the Docker ( Merkel, 2014) and Singularity ( Kurtzer *et al.*, 2017) containerisation solutions.

Table 4 contains a list of relevant software tool versions and sources.

**Table 4.** Software tools: versions and sources.

Software tool	Version	Source
BEDTools	2.30.0	https://github.com/arq5x/bedtools2
BLAST	2.14.0	ftp://ftp.ncbi.nlm.nih.gov/blast/executables/blast+/
BlobToolKit	4.3.9	https://github.com/blobtoolkit/blobtoolkit
BUSCO	5.5.0	https://gitlab.com/ezlab/busco
bwa-mem2	2.2.1	https://github.com/bwa-mem2/bwa-mem2
Cooler	0.8.11	https://github.com/open2c/cooler
DIAMOND	2.1.8	https://github.com/bbuchfink/diamond
fasta_windows	0.2.4	https://github.com/tolkit/fasta_windows
FastK	666652151335353eef2fcd58880bcef5bc2928e1	https://github.com/thegenemyers/FASTK
Gfastats	1.3.6	https://github.com/vgl-hub/gfastats
GoaT CLI	0.2.5	https://github.com/genomehubs/goat-cli
Hifiasm	0.19.5-r587	https://github.com/chhylp123/hifiasm
HiGlass	44086069ee7d4d3f6f3f0012569789ec138f42b84aa44357826c0b6753eb28de	https://github.com/higlass/higlass
MerquryFK	d00d98157618f4e8d1a9190026b19b471055b22e	https://github.com/thegenemyers/MERQURY.FK
Minimap2	2.24-r1122	https://github.com/lh3/minimap2
MitoHiFi	3	https://github.com/marcelauliano/MitoHiFi
MultiQC	1.14, 1.17, and 1.18	https://github.com/MultiQC/MultiQC
NCBI Datasets	15.12.0	https://github.com/ncbi/datasets
Nextflow	23.10.0	https://github.com/nextflow-io/nextflow
PretextView	0.2.5	https://github.com/sanger-tol/PretextView
purge_dups	1.2.5	https://github.com/dfguan/purge_dups
samtools	1.19.2	https://github.com/samtools/samtools
sanger-tol/ascc	-	https://github.com/sanger-tol/ascc
sanger-tol/blobtoolkit	0.5.1	https://github.com/sanger-tol/blobtoolkit
Seqtk	1.3	https://github.com/lh3/seqtk
Singularity	3.9.0	https://github.com/sylabs/singularity
TreeVal	1.2.0	https://github.com/sanger-tol/treeval
YaHS	1.2a.2	https://github.com/c-zhou/yahs

### Wellcome Sanger Institute – Legal and Governance

The materials that have contributed to this genome note have been supplied by a Darwin Tree of Life Partner. The submission of materials by a Darwin Tree of Life Partner is subject to the **‘Darwin Tree of Life Project Sampling Code of Practice’**, which can be found in full on the Darwin Tree of Life website here. By agreeing with and signing up to the Sampling Code of Practice, the Darwin Tree of Life Partner agrees they will meet the legal and ethical requirements and standards set out within this document in respect of all samples acquired for, and supplied to, the Darwin Tree of Life Project.

Further, the Wellcome Sanger Institute employs a process whereby due diligence is carried out proportionate to the nature of the materials themselves, and the circumstances under which they have been/are to be collected and provided for use. The purpose of this is to address and mitigate any potential legal and/or ethical implications of receipt and use of the materials as part of the research project, and to ensure that in doing so we align with best practice wherever possible. The overarching areas of consideration are:

•   Ethical review of provenance and sourcing of the material

•   Legality of collection, transfer and use (national and international)

Each transfer of samples is further undertaken according to a Research Collaboration Agreement or Material Transfer Agreement entered into by the Darwin Tree of Life Partner, Genome Research Limited (operating as the Wellcome Sanger Institute), and in some circumstances other Darwin Tree of Life collaborators.

## Data Availability

European Nucleotide Archive: Abax parallelepipedus. Accession number PRJEB64082; https://identifiers.org/ena.embl/PRJEB64082. The genome sequence is released openly for reuse. The *Abax parallelepipedus* genome sequencing initiative is part of the Darwin Tree of Life (DToL) project. All raw sequence data and the assembly have been deposited in INSDC databases. The genome will be annotated using available RNA-Seq data and presented through the Ensembl pipeline at the European Bioinformatics Institute. Raw data and assembly accession identifiers are reported in Table 1 and Table 2.

## Author information

Members of the Natural History Museum Genome Acquisition Lab are listed here: https://doi.org/10.5281/zenodo.12159242.

Members of the Darwin Tree of Life Barcoding collective are listed here: https://doi.org/10.5281/zenodo.12158331.

Members of the Wellcome Sanger Institute Tree of Life Management, Samples and Laboratory team are listed here: https://doi.org/10.5281/zenodo.12162482.

Members of Wellcome Sanger Institute Scientific Operations: Sequencing Operations are listed here: https://doi.org/10.5281/zenodo.12165051.

Members of the Wellcome Sanger Institute Tree of Life Core Informatics team are listed here: https://doi.org/10.5281/zenodo.12160324.

Members of the Tree of Life Core Informatics collective are listed here: https://doi.org/10.5281/zenodo.12205391.

Members of the Darwin Tree of Life Consortium are listed here: https://doi.org/10.5281/zenodo.4783558.
