# Supplementary material for: The genome sequence of a carabid beetle, Abax parallelepipedus (Piller & Mitterpacher, 1783)
Source: Wellcome Open Res. 2025 Mar 19;10:147. [Version 1] doi: 10.12688/wellcomeopenres.23888.1 (PMC12082080; doi:10.12688/wellcomeopenres.23888.1)
Supplement: Peer review history

# Reviewer report: Reviewer response for version 1

**DOI:** 10.21956/wellcomeopenres.26354.r121771

**Published:** 2025-05-14

**Authors:** Lapo Ragionieri

**Affiliations:** 1 Libera Universita di Bolzano Facolta di Scienze e Tecnologie, Bolzano, Trentino-Alto Adige/South Tyrol, Italy

The genome sequence of a carabid beetle, *Abax parallelepipedus* (Piller & Mitterpacher, 1783), by Olga Sivell, Duncan Sivell, Ryan Mitchell, and Maxwell Barclay describes the genome sequencing of a carabid species that is widespread across Europe and other continents. This species likely represents a species complex, and whole-genome sequencing will help clarify this aspect. The authors employed long-read sequencing technology (Pacific Biosciences single-molecule HiFi) to generate an initial assembly, which was then scaffolded using Hi-C, resulting in a chromosome-level genome assembly. Nearly all scaffolds were assigned to chromosomes, and the assembly also includes the mitochondrial genome. The genome assembly spans a total length of 567.62 Mb across 18 chromosomes.

 While the introduction and presentation of the results are well executed, the authors did not provide any information regarding genome masking. In general, all methods are well detailed and appear to be robust based on the metrics presented by the authors.

**Specific Comments and Suggestions:**

- **Keywords**: To be effective, keywords should differ from the title.
- **Annotation**: The authors should identify and quantify transposable elements within the genome and include this annotation in the current data note.
- **Command Lines**: It is standard practice to provide the command lines used for all software applications. Including this information would greatly benefit readers .

Are sufficient details of methods and materials provided to allow replication by others?

Yes

Is the rationale for creating the dataset(s) clearly described?

Yes

Are the datasets clearly presented in a useable and accessible format?

Yes

Are the protocols appropriate and is the work technically sound?

Yes

Reviewer Expertise:

genomics, transcriptomics, proteomics

I confirm that I have read this submission and believe that I have an appropriate level of expertise to confirm that it is of an acceptable scientific standard.

# Reviewer report: Reviewer response for version 1

**DOI:** 10.21956/wellcomeopenres.26354.r121769

**Published:** 2025-04-10

**Authors:** Michael G Ritchie

**Affiliations:** 1 University of St Andrews, Scotland, UK

This reports the sequence of a female of the carabid beetle *Abax parallelepipedus*. It is completed to a very high standard and the assembly, BUSCO and other stats are extremely impressive, even by the usual standard of the Tree of Life sequences. It is a good contribution to this project, and the data are freely available for downstream use.

**I have only minor comments for background information:**

- This is a female, but do we know if the species has an XY SD system or what? 
- How is the species distinguished from parallelus?
- The reference to 'unpublished records' from European Russia is unsupported. Should this be a 'pers comm' by ?
- The X chromosome assembly seems to have a very high similarity to Pterostichus. Is this a sibling species? How distinct is it, this seems eye-catching. 
- Add a reference for BOLD?

Are sufficient details of methods and materials provided to allow replication by others?

Yes

Is the rationale for creating the dataset(s) clearly described?

Yes

Are the datasets clearly presented in a useable and accessible format?

Yes

Are the protocols appropriate and is the work technically sound?

Yes

Reviewer Expertise:

Evolutionary genetics

I confirm that I have read this submission and believe that I have an appropriate level of expertise to confirm that it is of an acceptable scientific standard.
